# Prebiotic dietary fibers, gut microbiota, and short-chain fatty acid production: from fermentation mechanisms to clinical translation

**DOI:** 10.3389/fmicb.2026.1927734

**Published:** 2026-10-01

**Authors:** Kannadasan Charumathi, Seongok Kim, Hakdong Shin

**Affiliations:** 1 Carbohydrate Bioproduct Research Center & Department of Food Science and Biotechnology, Sejong University, Seoul, Republic of Korea; 2 Department of Food and Nutrition, College of BioNano Technology, Gachon University, Seongnam, Republic of Korea

**Keywords:** dietary fibers, gut microbiota, prebiotics, precision nutrition, SCFAs

## Abstract

The importance of the gut microbiota in regulating host homeostasis, immune responses, metabolism, and dietary interactions is increasingly recognized. Short-chain fatty acids (SCFAs), microbial metabolites produced through the fermentation of dietary substrates, play a central role in mediating these interactions. Drawing on mechanistic evidence from *in vivo* studies and insights from *in vitro* host–microbe fermentation models, this review critically examines how prebiotic dietary fibers have influenced the human gut microbial ecosystem, fermentation pathways, and SCFA production over the past decade. Major prebiotic classes, including fructooligosaccharides, galactooligosaccharides, inulin, resistant starches, pectins, xylooligosaccharides, human milk oligosaccharides, and selected polyphenols, are discussed in relation to their fermentability, microbial selectivity, and capacity to stimulate acetate, propionate, and butyrate production. Evidence from human intervention studies generally demonstrates modest yet biologically meaningful improvements in intestinal barrier integrity, inflammatory regulation, glucose homeostasis, lipid metabolism, and gut–brain communication. However, responses remain highly variable and are strongly influenced by baseline microbiota composition, habitual dietary patterns, medication exposure, intervention duration, and host metabolic status. Important gaps remain in linking mechanistic findings with clinical outcomes, explaining inter-individual differences in prebiotic responses, and standardizing methods for assessing microbiota and SCFAs. To address these limitations, this review proposes an integrated framework connecting prebiotic properties, microbial fermentation, SCFA-mediated host signaling, *in vitro* host–microbe models, and clinical evidence within the context of precision nutrition. This framework provides a systematic basis for understanding variable responses to prebiotics and for developing more personalized interventions. Future research should prioritize standardized analytical methods, well-designed stratified clinical trials, multi-omics integration, and computational approaches to identify responder phenotypes and optimize targeted prebiotic interventions.

## 1 Introduction

The gut microbiota has attracted increasing attention as a key determinant of host metabolism, immune function, and gastrointestinal homeostasis. Through the fermentation of undigested dietary substrates, the gut microbiota produces short-chain fatty acids (SCFAs), primarily acetate, propionate, and butyrate (Vinelli et al., 2022; Martin-Gallausiaux et al., 2021). These metabolites function as important signaling molecules that help maintain intestinal barrier integrity, regulate immune and inflammatory responses, modulate glucose and lipid metabolism, and influence gut–brain communication pathways. Consequently, dietary strategies aimed at enhancing SCFA production have generated considerable interest for their potential roles in disease prevention and therapeutic interventions.

Among dietary strategies, prebiotic dietary fibers are one of the most thoroughly investigated therapies for modifying the gut microbial flora. The International Scientific Association for Probiotics and Prebiotics (ISAPP) defines prebiotics as “substrates that are selectively utilized by host microbes and impart a health benefit” (Gibson et al., 2017; Hutkins et al., 2016). This category comprises non-digestible oligosaccharides, resistant polysaccharides, and plant-derived chemicals that escape digestion in the upper gastrointestinal tract and undergo microbial fermentation in the colon (Zhang et al., 2018). As illustrated in Figure 1, the selective fermentation of prebiotic substrates promotes cross-feeding interactions among microbes and the production of short-chain fatty acids (SCFAs), leading to various beneficial health outcomes. These microbial and metabolic interactions influence both intestinal and systemic physiology by selectively enriching beneficial microbial populations and modifying microbial metabolic activity. According to emerging evidence, prebiotics supplementation may improve immunological, cardiovascular, metabolic, and neurological processes in addition to the gastrointestinal tract (Jenkins and Mason, 2022). Prebiotic use may help prevent colorectal cancer, lessen the symptoms of irritable bowel syndrome (Yassine et al., 2025), and lower levels of total cholesterol and triacylglycerol (TAG). Furthermore, prebiotics may increase the activity of natural killer (NK) cells and regulate the production of cytokines, such as IL-4, IL-8, and IL-10, albeit these effects differ according on the prebiotic substrate, research population, and clinical setting (Vulevic et al., 2015). Although these findings revealed the wide range of medical benefits of prebiotics, there are still important questions about how they work, their consistency, and individual variations.

**Figure 1:**
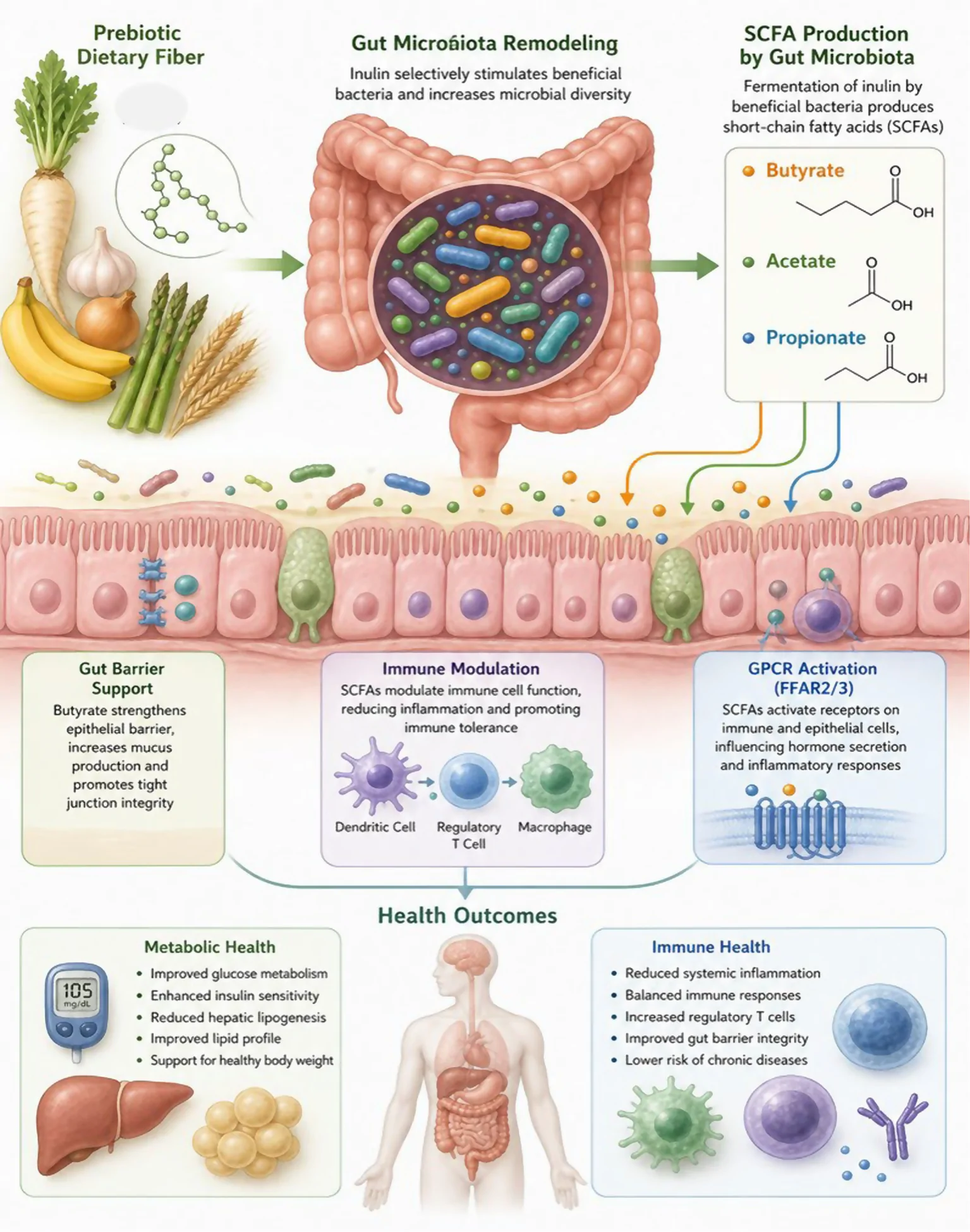
Integrative framework linking prebiotic substrate classes, gut microbial fermentation, SCFA biosynthetic pathways, and host physiological outcomes.

Over the past decade, research on prebiotic-mediated gut microbiota modulation has evolved through three major stages. Early investigations primarily characterized fermentable dietary substrates and their selective effects on beneficial microbial taxa. Subsequent studies elucidated microbial fermentation pathways and the biosynthesis of SCFAs, establishing acetate, propionate, and butyrate as central mediators of host–microbe interactions involved in epithelial homeostasis, immune regulation, metabolic control, and gut–brain communication. More recently, advances in multi-omics technologies, sophisticated *in vitro* host–microbe models, and precision nutrition have shifted attention toward understanding individualized responses to prebiotic interventions and translating microbiome science into clinical practice.

Despite these advances, the current evidence remains fragmented. Most studies examine individual aspects of prebiotic biology, such as substrate fermentation, microbial ecology, SCFA signaling, experimental model development, or disease-specific interventions. However, relatively few studies integrate these processes within a unified translational framework (Vinelli et al., 2022; Van-Wehle and Vital, 2024; Wang et al., 2024). Consequently, three major knowledge gaps remain. First, direct comparative studies of the fermentation characteristics, microbial selectivity, and SCFAs production profiles of different prebiotic substrates remain limited. Second, although the signaling pathways of individual SCFAs have been extensively investigated, their integration into a systems-level framework linking microbial metabolic changes to host physiology remains incomplete. Third, the mechanisms underlying inter-individual variation in responses to prebiotic interventions remain poorly understood. Potential determinants include baseline gut microbiota composition, habitual diet, host metabolic status, medication use, analytical methodology, and study design.

Addressing these challenges requires an integrated analytical framework capable of connecting microbial ecology, fermentation metabolism, host signaling, experimental validation, and clinical translation. To bridge these knowledge gaps, this review follows a step-by-step analytical framework based on a complete biological continuum ranging from food components to medicinal applications. First, the functional classification and fermentation characteristics of key prebiotic fibers are examined. This explains how the structure of food components influences how microbes utilize them and determines their fermentation potential. Next, microbial fermentation ecology and the biochemical pathways for SCFAs production are explored. This clarifies the metabolic processes responsible for the formation of SCFAs such as acetate, propionate, and butyrate. Based on these mechanistic fundamentals, this review brings together the current knowledge on the physiological functions influenced by SCFAs. This includes regulation of the gut epithelial barrier, modulation of immune responses, maintenance of metabolic homeostasis, and gut–brain communication. These mechanistic findings are then evaluated using advanced *in vitro* host–microbe co-culture systems and experimental models before translation to human studies.

This review critically examines evidence from intervention studies conducted in humans. It evaluates how prebiotics induce changes in the gut microbiota and influence metabolic, inflammatory, oncological, and neurophysiological outcomes, as well as the clinical significance of these effects. Finally, the various pieces of evidence gathered are integrated under the emerging concept of precision prebiotic nutrition. Here, multi-omics technologies, computational predictions, and individual gut microbiota responses to prebiotics play key roles. These help in developing personalized dietary interventions targeting the microbiota. The resulting analytical sequence is as follows: classification of primary components → microbial colonization and metabolic changes → SCFA-mediated host signaling → *in vitro* model verification → human clinical evidence → practical implementation of precision nutrition. By following this analytic sequence, this review provides an integrated systems-level perspective. It connects fundamental microbial processes with medical applications and offers a broad conceptual framework for future research on gut microbiota-targeted nutrition and the development of personalized treatment strategies.

## 2 Literature search strategy

A narrative literature review was conducted to synthesize recent findings on prebiotic dietary fibers, gut microbiota regulation, and short-chain fatty acid (SCFA) production. Literature searches were performed using the PubMed, Scopus, Web of Science, and Google Scholar databases, with primary emphasis on research articles published between 2015 and 2025 to capture recent advances in prebiotic fermentation mechanisms, gut microbiota modulation, SCFA biology, experimental models, and clinical intervention research. Foundational studies published before 2015, including the original conceptual framework of prebiotics (Gibson and Roberfroid, 1995; Gibson et al., 2010), the updated ISAPP definition of prebiotics (Gibson et al., 2017), landmark investigations of SCFA-mediated host signaling (Louis et al., 2014; Koh et al., 2016), and pioneering work on gut microbial ecology and host–microbe interactions (Flint et al., 2012), were incorporated through manual reference tracing of key reviews and highly cited publications. This strategy ensured that the review balanced canonical theoretical concepts with the latest mechanistic discoveries and translational evidence. The main search keywords used were prebiotic dietary fiber, gut microbiota, SCFA, inulin, fructooligosaccharides, galactooligosaccharides, resistant starch, intestinal barrier, gut–brain axis, host–microbe interaction, precision nutrition, and *in vitro* fermentation models. Additionally, the reference lists of selected studies were screened to identify relevant publications. Human clinical studies, systematic reviews, meta-analyses, and mechanistic studies were prioritized. Animal studies and laboratory-based (*in vitro*) research that provided important information on microbial colonization, SCFA production, and host–microbe interactions were included. Studies related to prebiotic fermentation, changes in gut microbiota, the effects of SCFAs, and their applications for human health were selected. Since this is a narrative review, no risk-of- bias assessment or quantitative meta-analysis was performed to formally evaluate the quality of the studies.

## 3 Classification and fermentation characteristics of prebiotic fibers

Plant-derived polysaccharides, oligosaccharides, and other indigestible carbohydrates that are resistant to digestion in the upper gastrointestinal tract and arrive mostly undigested in the colon, where they act as substrates for microbial fermentation, are referred to as dietary fibers (Munteanu and Schwartz, 2024; Deehan et al., 2024). Short-chain fatty acids (SCFAs) and other bioactive metabolites that impact intestinal and systemic physiology are produced by microbial metabolism of these substances (Martin-Gallausiaux et al., 2021).

Importantly, not all dietary fibers qualify as prebiotics. According to the SAPP, prebiotics are “substrates that are selectively utilized by host microorganisms and confer a health benefit” (Gibson et al., 2017). To meet this criterion, compounds must be resistant to host digestion, metabolized by the gut microbiota, and selectively stimulate beneficial microbial taxa or functions associated with enhanced host health (Gibson et al., 2017; Hutkins et al., 2016). The physiological significance of prebiotic fibers goes beyond their traditional effects on gut function. By promoting saccharolytic fermentation and reducing proteolytic metabolism, prebiotics contribute to intestinal barrier integrity, immune regulation, glucose homeostasis, lipid metabolism, and gut–brain communication. Prebiotic intake has been related to improved metabolic and inflammatory outcomes in clinical and experimental investigations, albeit effects vary greatly depending on fiber structure, dosage, regular diet, and baseline microbiota composition (Deehan et al., 2024; Delzenne et al., 2025). There is growing evidence that microbial functional ability rather than microbial numbers alone mostly determine response to prebiotic therapies.

### 3.1 Functional classification of fermentable prebiotic fibers

The physiological effects of prebiotics are strongly influenced by substrate structure, accessibility, and availability. Accordingly, contemporary classification approaches increasingly consider fermentability, degree of polymerization (DP), molecular complexity, branching, glycosidic linkages, physicochemical properties, and microbial selectivity rather than relying solely on conventional soluble-insoluble fiber categories. These features influence microbial enzyme accessibility, fermentation’s kinetics, the spatial distribution of fermentation along the colon, and the resulting metabolic profile (Deehan et al., 2017; Van-Wehle and Vital, 2024).

Slowly degradable polysaccharides and resistant starches can sustain fermentation into more distal regions of the colon and are frequently associated with enhanced butyrate production. In contrast, rapidly fermentable oligosaccharides, such as fructooligosaccharides (FOS) and galactooligosaccharides (GOS), are generally metabolized earlier in the colon and commonly promote acetate and lactate production, which can subsequently support propionate- and butyrate-producing microorganisms through microbial cross-feeding. Importantly, SCFA production is not a fixed property of individual prebiotics but emerges from interactions among substrate structure, dose, fermentation kinetics, baseline microbiota composition, cross-feeding networks, and host-specific factors.

Table 1 summarizes the major structural and fermentation characteristics of commonly studied prebiotic substrates, linking molecular architecture with fermentation behavior, predominant SCFA profiles, and representative microbial taxa. FOS and GOS are generally rapidly fermented and frequently promote *Bifidobacteria*, particularly members of the genus *Bifidobacterium*. Their primary fermentation products, including acetate and lactate, can subsequently serve as substrates for other microorganisms, contributing to secondary fermentation and butyrate production through cross feeding (Roberfroid et al., 2010; Holscher, 2017). Inulin-type fructans similarly exert bifidogenic effects, although their fermentation kinetics and microbial responses vary according to chain length and molecular structure (Le Bastard et al., 2020). These differences illustrate how molecular architecture can influence substrate utilization and downstream microbial metabolism.

**Table 1:** Structural characteristics, fermentation behavior, SCFA production profiles, and microbiota modulation by major prebiotic substrates.

Prebiotic substrate	Degree of polymerization (DP)	Branching/structural characteristics	Major glycosidic linkages	Crystallinity/physicochemical characteristics	Predominant fermentation location	Typical SCFA production profile*	Representative microbial taxa	References
Fructooligosaccharides (FOS)	~DP 2–9	Mainly short, relatively linear fructan chains	Predominantly β-(2 → 1); β-(2 → 6) may occur depending on FOS type	Highly soluble; relatively low structural order	Mainly proximal colon; rapid fermentation	Acetate dominant; propionate and butyrate variable	*Bifidobacterium, Bacteroides, Faecalibacterium*	Roberfroid et al. (2010), Holscher (2017)
Galactooligosaccharides (GOS)	~DP 2–8	Short chains; branching and linkage patterns depend on synthesis conditions	Mainly β-(1 → 4), β-(1 → 6), and β-(1 → 3)	Highly soluble; generally amorphous	Mainly proximal colon	Acetate dominant, with propionate and butyrate generated through fermentation and cross-feeding	*Bifidobacterium, Bacteroides, Lactobacillus*	Roberfroid et al. (2010), Holscher (2017)
Inulin	~DP 2–60+; highly source-dependent	Predominantly linear fructan; limited branching may occur	Mainly β-(2 → 1) fructosyl linkages	Semi-crystalline; properties strongly affected by DP and processing	Proximal → distal colon; longer-chain fractions may ferment more slowly	Acetate + propionate, with butyrate promoted through cross-feeding	*Bifidobacterium, Bacteroides, Faecalibacterium, Roseburia*	Roberfroid et al. (2010), Holscher (2017), Le Bastard et al. (2020)
Resistant starch (RS)	Highly variable; long glucose polymers	Linear and/or α-(1 → 6)-branched depending on RS type	α-(1 → 4), α-(1 → 6)	Relatively high structural order; crystallinity, amylose content, retrogradation, and accessibility influence resistance	Proximal → distal colon; relatively slow fermentation	Acetate + butyrate; frequently butyrogenic, depending on RS type and microbiota	*Ruminococcus bromii, Bifidobacterium adolescentis, Eubacterium rectale, Roseburia, Faecalibacterium*	Martin-Gallausiaux et al. (2021), Deehan et al. (2024), Jung and Park (2023)
Pectin	Highly heterogeneous; polysaccharide-scale	Homogalacturonan with branched rhamnogalacturonan-I/II domains	Mainly α-(1 → 4)-galacturonosyl; additional α- and β-linked side-chain residues	Hydrated, gel-forming and structurally heterogeneous; affected by molecular weight and degree of esterification	Mainly colon; fermentation may extend toward distal regions	Acetate generally dominant; propionate and butyrate variable	*Bacteroides, Lachnospira, Faecalibacterium, other pectinolytic taxa*	Larsen et al. (2019), Pascale et al. (2022), Cao et al. (2024)
Human milk oligosaccharides (HMO)	~DP 3–30+	Highly diverse, linear and branched; frequently fucosylated and/or sialylated	β-(1 → 3), β-(1 → 4), β-(1 → 6), α-(1 → 2), α-(1 → 6), depending on HMO	Highly soluble; complex glycan architecture	Primarily proximal colon in infants	Acetate dominant, with lactate and downstream SCFAs generated through cross-feeding	*Bifidobacterium longum subsp. infantis, B. bifidum, B. breve*	Thomson et al. (2018), Lordan et al. (2024)
Polyphenols/polyphenol-derived substrates	Not applicable as a uniform DP parameter; monomers, oligomers, and polymers occur	Highly diverse aromatic structures; polymerization varies	Variable O-/C-glycosidic and ester linkages	Highly variable; influenced by molecular size, polymerization, and food matrix	Small intestine → colon; poorly absorbed compounds undergo microbial transformation	Highly variable; acetate, propionate and/or butyrate may increase	*Bifidobacterium, Akkermansia, Faecalibacterium, Roseburia*	Ozdal et al. (2016), Plamada and Vodnar (2021)

SCFA profiles represent general tendencies rather than fixed stoichiometric ratios. Actual acetate, propionate, and butyrate production depends on substrate structure, concentration, fermentation kinetics, donor microbiota, cross-feeding, and host-specific factors.

Resistant starch is particularly relevant to butyrate-oriented microbial networks because it escapes digestion in the small intestine and undergoes gradual degradation in the colon. Specialized amylolytic microorganisms initiate this process, with *Ruminococcus bromii* considered an important keystone species in resistant-starch degradation. Secondary fermenters, supporting butyrate-producing taxa such as *Eubacterium rectale*, *Roseburia*, and *Faecalibacterium* (Martin-Gallausiaux et al., 2021; Deehan et al., 2024), can subsequently utilize the resulting degradation products. However, the magnitude of butyrate production depends on the functional capacity and composition of the resident microbial community rather than on resistant starch alone.

Pectins constitute a structurally heterogeneous group of plant polysaccharides whose microbial utilization is influenced by molecular weight, degree of methyl esterification, branching, and the relative abundance of homogalacturonan and rhamnogalacturonan domains. Their structural complexity permits sequential degradation by multiple microbial taxa and facilitates cross-feeding interactions. Pectin fermentation can therefore contribute to acetate, propionate, and butyrate production, although the magnitude and relative proportions of these metabolites vary according to substrate structure and microbial community characteristics (Larsen et al., 2019; Pascale et al., 2022; Cao et al., 2024). Pectin should consequently be regarded as a structurally diverse substrate rather than a chemically uniform prebiotic.

Human milk oligosaccharides (HMOs) represent another structurally diverse group of microbiota-directed substrates. Their utilization depends on specific glycosidases and carbohydrate-utilization systems present in infant-associated microbial taxa. *Bifidobacterium longum* subsp. infantis, *B. bifidum*, and *B. breve* possess distinct capacities for HMO utilization, enabling selective microbial enrichment and production of acetate and other fermentation metabolites. These metabolites can subsequently participate in cross-feeding interactions with other members of the infant gut microbiota (Thomson et al., 2018; Lordan et al., 2024). Although HMOs are primarily recognized for their role in infant nutrition and microbiome development, their microbiota-directed properties have also generated interest in targeted microbiota modulation and precision nutrition (Okburan and Kızıler, 2023). Overall, prebiotic effects reflect interactions among substrate structure, microbial functional capacity, and fermentation environment, collectively shaping SCFA profiles and supporting microbiome-informed precision nutrition.

### 3.2 Emerging non-carbohydrate prebiotic modulators

Beyond carbohydrate-based compounds, there is growing interest in non-carbohydrate food components, particularly polyphenols, that influence the composition and functional activity of gut microbiota. Although polyphenols are not universally classified as prebiotics under the current ISAPP definition, several polyphenolic compounds exhibit prebiotic-like or microbiota-modulating effects through selective stimulation or inhibition of microbial taxa, modification of microbial metabolic pathways, and microbial transformation into bioactive metabolites (Gibson et al., 2017; Ozdal et al., 2016). Polyphenolic compounds like cocoa flavanols, quercetin, resveratrol, and curcumin not only promote interactions between microbes through microbial cross feeding but also support the growth of beneficial microbial species. They also boost saccharolytic fermentation and help control the growth of microbes associated with gut microbiota imbalance (dysbiosis) (Ozdal et al., 2016). Further studies suggest that quercetin may strengthen intestinal function, modulate gut microbiota, and attenuate metabolic and inflammatory disturbances associated with obesity (Zhao et al., 2018; Shabbir et al. 2021).

In addition to their chemical classification, prebiotic and prebiotic-like substrates’ biological effects are influenced by their molecular structure, fermentability, microbial selectivity, substrate accessibility and cooperative microbial metabolism (Deehan et al., 2020; Van-Wehle and Vital, 2024). These factors collectively determine which microbial populations can access a substrate, where fermentation occurs, which metabolic intermediates are generated, and how cross-feeding ultimately shapes the SCFA profile.

Finally, the ecological and metabolic characteristics of the gut microbiome determine how dietary substrates are converted into metabolites capable of influencing host physiology. As illustrated in Figure 1, these processes involve interconnected pathways linking dietary substrate consumption, microbial degradation, primary and secondary fermentation, SCFA generation, microbial cross-feeding, and host responses (Martin-Gallausiaux et al., 2021; Van-Wehle and Vital, 2024). This framework provides a mechanistic bridge between the structural properties of prebiotic substrates, microbial fermentation ecology, SCFA-mediated host signaling, and their potential clinical and physiological consequences.

## 4 Microbial fermentation of dietary substrates and ecological determinants of SCFA production

Short-chain fatty acid (SCFA) production is predominantly driven by bacterial fermentation of non-digestible carbohydrates that escape digestion in the upper gastrointestinal tract and enter the colon. Resistant starches (RS), inulin-type fructans, galactooligosaccharides (GOS), arabinoxylans, pectin’s, and other carbohydrates accessible to the microbiota are among the most common fermentable substrates (Macfarlane and Macfarlane, 2012; Deehan et al., 2024). Although protein fermentation contributes to colonic metabolism, it produces fewer SCFAs and generates metabolites such as branched-chain fatty acids, ammonia, phenols, and indoles, which may have distinct physiological effects depending on their concentrations and exposure conditions (Macfarlane and Macfarlane, 2012; Mao et al., 2023).

Human digestive enzymes can only hydrolyze a limited number of glycosidic linkages found in complex plant polysaccharides. Consequently, many dietary fibers reach the colon mainly intact, where their breakdown is dependent on microbial carbohydrate-active enzymes (CAZymes) (Cantarel et al., 2009; Wardman et al., 2022). These enzymes, which include glycoside hydrolases (GHs), polysaccharide lyases (PLs), carbohydrate esterases (CEs), and carbohydrate-binding modules (CBMs), which facilitate the recognition and degradation of complex carbohydrates into fermentable oligosaccharides and monosaccharides. These products subsequently fuel microbial fermentation and contribute to SCFA production (Wardman et al., 2022).

Importantly, the capacity of gut microorganisms to degrade dietary fiber varies considerably among species and, even among strains within the same species, owing to differences in their carbohydrate-active enzyme (CAZyme) repertoires, polysaccharide utilization loci (PULs), carbohydrate transport systems, and downstream metabolic pathways (Onyango et al., 2021; Wardman et al., 2022; Qu et al., 2025). For example, *Ruminococcus bromii* functions as a keystone degrader of resistant starch, initiating substrate breakdown and releasing fermentation intermediates that can subsequently be utilized by butyrate-producing microorganisms, thereby facilitating cross-feeding interactions that promote butyrate production In contrast, members of the genera *Bacteroides* and *Parabacteroides* possess extensive polysaccharide utilization loci (PULs) that enable the degradation of a broad range of plant-derived glycan’s (Wardman et al., 2022; Qu et al., 2025). *Bifidobacterium* species possess specialized glucosidases and carbohydrate-utilization pathways that facilitate the metabolism of oligosaccharides such as fructooligosaccharides (FOS) and galactooligosaccharides (GOS) and, particularly in infants, human milk oligosaccharides (HMOs). These examples illustrate that microbial functional capacity—including CAZyme repertoires, PULs, substrate transport systems, and downstream metabolic pathways—may provide a more informative predictor of fermentation outcomes than taxonomic composition alone.

Numerous ecological and host-related factors can have an impact on SCFA production. Throughout life, fermentation patterns are altered by age-related changes in microbiota composition. While adult microbiomes typically show a greater capacity for butyrate and propionate production from complex dietary fibers, infant microbiomes enriched in *Bifidobacterium* species preferentially produce acetate through fermentation of human milk oligosaccharides (Dinleyici et al., 2023; Van-Wehle and Vital, 2024). SCFA concentrations are also influenced by spatial variation within the colon; fermentation activity usually peaks in the proximal colon, where substrate availability is highest, and decreases in distal parts due to substrate depletion and SCFA absorption (Louis and Flint, 2017).

To further illustrate the functional differences among major microbial groups involved in dietary fiber fermentation, Table 2 summarizes their representative CAZyme characteristics, preferred substrates, and contributions to SCFA production. The comparison in Table 2 illustrates an important distinction between taxonomic composition and microbial functional capacity. Two individuals may possess broadly similar bacterial genera but differ substantially in the abundance and activity of specific CAZymes, PULs, carbohydrate transporters, and SCFA biosynthetic pathways. Conversely, taxonomically different microbial communities may exhibit similar metabolic functions when they possess overlapping carbohydrate-degradation and fermentation capabilities. Thus, the presence or abundance of a bacterial genus alone provides limited information about its actual contribution to SCFA production.

**Table 2:** Major microbial groups, CAZyme functions, substrate preferences, and contributions to SCFA production.

Microbial group	Representative bacteria	Main CAZyme/functional features	Major substrate preferences	Main SCFA contribution	References
Butyrate producers	*Faecalibacterium, Roseburia, Eubacterium, Anaerobutyricum*	Glycoside hydrolases; carbohydrate transporters; butyrate-synthesis enzymes	Resistant starch, arabinoxylans, β-glucans, acetate and lactate	Butyrate; often utilize acetate/lactate from other microbes	Duncan et al. (2002), Rivière et al. (2016)
Bifidobacteria	*B. longum, B. adolescentis, B. breve, B. bifidum*	Diverse glycosidases and oligosaccharide-utilization enzymes	FOS, GOS, inulin, HMO	Acetate + lactate; supports secondary butyrate production	Belenguer et al. (2006), Thomson et al. (2018), Liu et al. (2017)
Bacteroides/Parabacteroides	*B. thetaiotaomicron, B. ovatus, B. uniformis*	Extensive CAZymes and polysaccharide-utilization loci (PULs)	Pectin, arabinoxylan, hemicellulose, fructans, complex plant polysaccharides	Acetate + propionate; provides substrates for cross-feeding	Martens et al. (2011), Wardman et al. (2022)
Resistant-starch degraders	*Ruminococcus bromii, B. adolescentis*	Starch-degrading CAZymes and carbohydrate-binding systems	Resistant starch	Mainly fermentation intermediates; promotes downstream butyrate	Ze et al. (2012), Deehan et al. (2024)
Lactate-producing fermenters	*Bifidobacterium, Lactobacillus*	Glycosidases and carbohydrate-fermentation pathways	GOS, FOS and readily fermentable carbohydrates	Lactate + acetate; lactate supports butyrate-producing bacteria	Belenguer et al. (2006), Rivière et al. (2016)

CAZymes, carbohydrate-active enzymes; FOS, fructooligosaccharides; GOS, galactooligosaccharides; HMO, human milk oligosaccharides; PULs, polysaccharide utilization loci; SCFAs, short-chain fatty acids. Note: CAZyme repertoires and substrate utilization vary substantially among species and strains; therefore, the table summarizes general functional tendencies rather than universal characteristics of each microbial group.

This distinction is particularly important when interpreting responses to prebiotic supplementation. An increase in *Bifidobacterium*, abundance does not necessarily translate into a proportional increase in butyrate production because many *Bifidobacteria* species primarily produce acetate and lactate. Butyrate formation may instead depend on the presence of organisms capable of converting these metabolites through cross-feeding pathways. Similarly, the abundance of *Bacteroides* does not necessarily predict a specific SCFA profile, as different species and strains possess distinct poly utilization loci (PULs) and carbohydrate-active enzyme (CAZyme) repertoires, resulting in differences in their capacity to degrade and utilize specific polysaccharides (Martens et al., 2011; Wardman et al., 2022). Therefore, SCFA production is better understood as an emergent property of interconnected microbial metabolic network rather than as a direct consequence of the abundance of individual taxa.

### 4.1 Functional genomes as determinants of SCFA production

The functional-genome perspective provides a mechanistic framework for understanding inter-individual variability in prebiotic responses. Microbial genomes encode the enzymes, transporters, and metabolic pathways required to degrade specific dietary substrates. CAZyme abundance and diversity determine fiber-degradation capacity, whereas downstream metabolic pathways influence the conversion of fermentation products into acetate, propionate, butyrate, lactate, and other metabolites (Cantarel et al., 2009; Wardman et al., 2022).

Thus, the same prebiotic can produce different SCFA responses among individuals. Although two individuals may harbor similar bacterial taxa, differences in polysaccharide utilization loci (PULs), CAZyme profiles, metabolic pathways, and cross-feeding networks can alter their capacity to utilize pectin, arabinoxylan, resistant starch, or fructans. Similarly, increased bifidobacteria following FOS or GOS supplementation does not necessarily result in equivalent butyrate production because downstream conversion depends on compatible cross-feeding partners.

### 4.2 Ecological and host determinants of SCFA production

SCFA production is influenced by multiple factors, including age, gastrointestinal region, gut microbiota composition, diet, and host physiology. In children, the gut microbiota contains a greater abundance of *Bifidobacterium* species capable of utilizing human milk oligosaccharides (HMOs), contributing to higher acetate production. In contrast, the adult gut microbiota contains diverse primary and secondary fermenters that utilize complex dietary fibers to produce propionate and butyrate (Dinleyici et al., 2023; Van-Wehle and Vital, 2024). Fermentation is generally greatest in the proximal colon because carbohydrate availability is higher, whereas substrate depletion, microbial adaptation, SCFA absorption, and changing luminal conditions influence fermentation distally (Louis and Flint, 2017). Importantly, lower distal-colonic SCFA concentrations do not necessarily indicate reduced production because colonocytes and other host tissues absorb substantial SCFAs.

Because gut microbiome composition varies among individuals, microbial metabolite production also varies. Rather than being classified into discrete types called “enterotypes,” gut microbial communities are increasingly viewed as varying along continuous compositional gradients. Communities enriched in *Bacteroides* or *Prevotella* may differ in their capacity to degrade carbohydrates and produce metabolites (Costea et al., 2018). Together with these microbial differences, diet, gut transit time, luminal pH, medication use, age, inflammation, and host metabolic status contribute to inter-individual variation in nutrient metabolism and SCFA production (Blaak et al., 2020; Louis and Flint, 2017).

### 4.3 Critical perspective: from taxonomic associations to functional prediction

A key limitation of current prebiotic research is its reliance on taxonomic abundance as an indicator of microbial function. Although 16S rRNA sequencing can identify broad compositional changes, it cannot determine metabolic activity or whether organisms possess the enzymatic machinery required to utilize a particular substrate. Taxonomic enrichment therefore does not necessarily indicate enhanced fermentation or SCFA production.

A more mechanistic approach should integrate metagenomics, CAZyme profiling, metatranscriptomics, metabolomics, and direct SCFA measurements. Metagenomics identifies genetic potential, metatranscriptomics assesses pathway expression, and metabolomics determines whether functional changes translate into altered SCFA and intermediate-metabolite production. This integration is essential because gene abundance does not necessarily reflect gene expression or metabolic flux.

Moreover, fecal SCFA concentrations are not direct measures of total colonic SCFA production because SCFAs are rapidly absorbed and metabolized by colonocytes and the liver. Fecal concentrations therefore reflect the balance among microbial production, luminal utilization, epithelial uptake, and host metabolism. Future studies should distinguish among microbial production capacity, luminal SCFA concentrations, and systemic SCFA exposure when assessing the effects of prebiotics.

Overall, SCFA production results from a dynamic network involving substrate availability, CAZyme-mediated degradation, primary fermentation, microbial cross-feeding, secondary fermentation, SCFA production, and host uptake. This functional framework better explains inter-individual variability than taxonomy alone and supports the development of precision prebiotic nutrition based on microbial functional capacity.

## 5 SCFA-mediated host signaling

SCFAs are critical signaling molecules that connect prebiotic metabolism with immunological, endocrine, epithelial, and systemic physiological responses. Table 3 summarizes the major biological activities of acetate, propionate, and butyrate, highlighting their distinct metabolic fates and their complementary roles in intestinal barrier integrity, immune regulation, enteroendocrine signaling, and systemic energy metabolism. Specialized intestinal microbiota ferment prebiotic substrates and generate acetate, propionate, and butyrate through interconnected fermentation and cross-feeding pathways. These short-chain fatty acids differ in their abundance, tissue distribution, receptor affinity, and metabolic fate. Importantly, their physiological effects depend not only on microbial production but also on epithelial absorption, colonocyte utilization, portal transport, and hepatic metabolism, highlighting the complex pathways through which prebiotic consumption influences host health (Koh et al., 2016; Blaak et al., 2020; Mann et al., 2024). As a result, fecal SCFA concentrations should be interpreted as markers of microbial fermentation activity rather than direct measures of systemic SCFA exposure, because most SCFAs are absorbed and metabolized before excretion.

**Table 3:** Biological activities and host signaling functions of major short-chain fatty acids (SCFAs).

SCFAs	Biological activities	References
Acetate	Most abundant circulating SCFA; serves as a peripheral substrate for lipogenesis, cholesterol synthesis, skeletal muscle oxidation, and enteroendocrine receptor signaling; contributes to appetite regulation and maintenance of intestinal microbial balance.	Liu et al. (2018), Portincasa et al. (2022), Wang et al. (2024)
Propionate	Primarily metabolized in the liver where it supports gluconeogenesis, hepatic lipid regulation, cholesterol lowering, and stimulation of GLP-1 and PYY secretion; associated with satiety promotion and anti-inflammatory metabolic signaling.	Haghikia et al. (2015), Martin-Gallausiaux et al. (2021), Portincasa et al. (2022)
Butyrate	Primary oxidative energy source for colonocytes; enhances epithelial tight junction integrity, suppresses intestinal inflammation via HDAC inhibition and regulatory T-cell induction, and exerts anti-proliferative actions protective against colorectal carcinogenesis.	Hajjar et al. (2021), Salvi and Cowles (2021), Gustafsson and Johansson (2022)
Acetate, propionate and butyrate	Collectively regulate intestinal epithelial renewal, mucosal immunity, cytokine balance, enteroendocrine hormone release, host energy harvesting, and gut-brain communication; implicated in metabolic syndrome prevention, inflammatory control, and reduced colorectal disease risk.	Martin-Gallausiaux et al. (2021), Salvi and Cowles (2021), Wang et al. (2024)

### 5.1 Cellular uptake and molecular signaling pathways

SCFAs exert diverse biological effects through both receptor-dependent and receptor-independent mechanisms. Acetate, propionate, and butyrate activate G-protein-coupled receptors on the cell surface, including FFAR2 (GPR43), FFAR3 (GPR41), and GPR109A, which are found on intestinal epithelial, entero-endocrine, immunological, and adipose cells (Moniri and Farah, 2021; Mann et al., 2024). In addition to receptor-mediated signaling, SCFAs enter cells via passive diffusion and monocarboxylate transporters such SLC16A1 and SLC5A8. Butyrate and propionate, once internalized, inhibit histone deacetylase (HDAC) activity, promoting histone acetylation and modulating gene expression programs involved in epithelial integrity, immune regulation, cellular differentiation, and metabolism (Parada Venegas et al., 2019; Portincasa et al., 2022). Together, membrane receptor activation and epigenetic regulation enable SCFAs to translate microbial activity into coordinated host responses. These molecular signaling pathways underpin the diverse physiological effects of SCFAs on intestinal barrier function, immune regulation, and systemic metabolism.

### 5.2 Intestinal barrier function and immune regulation

Among SCFAs, butyrate plays a particularly important role in maintaining intestinal barrier integrity. It promotes tight junction assembly, mucin synthesis, and epithelial defense mechanisms, reducing microbial translocation and maintaining mucosal homeostasis (Parada Venegas et al., 2019; Mann et al., 2024). Furthermore, SCFAs lower luminal pH, promoting saccharolytic microbes while inhibiting the growth of potentially harmful species. Enrichment of SCFA-producing taxa such as *Akkermansia muciniphila, Bifidobacterium longum, Eubacterium rectale, Faecalibacterium prausnitzii* and *Roseburia intestinalis* has been linked to improved barrier function and immune homeostasis (Parada Venegas et al., 2019; Liu et al., 2018). SCFAs also have a broad immunomodulatory effect, regulating dendritic cells, macrophages, neutrophils, and T lymphocytes. Butyrate and propionate stimulate regulatory T-cell differentiation, increase IL-10 signaling, and help limit excessive inflammatory responses. However, these effects are context-dependent and may differ depending on SCFA concentration, illness status, and local cytokine settings (Furusawa et al., 2013; Mann et al., 2024). Thus, SCFAs act as immunoregulatory rather than anti-inflammatory metabolites.

### 5.3 Energy homeostasis and endocrine signaling

SCFAs contribute to host energy metabolism by enabling microbes to recover energy from otherwise indigestible carbohydrates, resulting in an estimated 600–750 kJ of usable energy per day (Blaak et al., 2020). Their physiological significance, however, goes beyond energy recovery. Butyrate is the primary oxidative fuel for colonocytes, while propionate is transferred to the liver and contributes to gluconeogenesis and lipid metabolism. Peripheral tissues use acetate, the most abundant circulating SCFS, for oxidative metabolism and lipogenesis (Koh et al., 2016; Boets et al., 2017). In addition to their metabolic functions, SCFA serve as signaling molecules that impact enteroendocrine activity. Activation of FFAR2 and FFAR3 on enteroendocrine L-cells promotes the production of glucagon-like peptide-1 (GLP-1) and peptide YY (PYY), which regulate appetite, insulin sensitivity, gastric emptying, and postprandial glucose homeostasis (Tolhurst et al., 2012). As a result, microbial fermentation has a direct impact on overall metabolic control. SCFAs play further roles in modifying obesity, insulin resistance, hepatic steatosis, and chronic low-grade inflammation, however responses are still highly personalized (Blaak et al., 2020; Canfora et al., 2019).

### 5.4 Epigenetic regulation: a key mechanism of SCFA action

Beyond acute receptor-mediated signaling, SCFAs have long-term biological impacts via epigenetic regulation. Butyrate, in particular, inhibits histone deacetylase (HDAC), changing gene expression in a variety of host tissues. HDAC inhibition increases the expression of tight junction proteins like claudin-1 and occludin, promotes FOXP3 transcription and regulatory T-cell differentiation, and regulates cytokine production by suppressing pro-inflammatory mediators and increasing anti-inflammatory signals like interleukin-10 (Furusawa et al., 2013; Parada Venegas et al., 2019; Salvi and Cowles, 2021). These findings identify SCFAs as key microbiota-derived epigenetic regulators capable of changing transcriptional programs involved in epithelial maintenance, immunological tolerance, and metabolic balance (Figure 2).

**Figure 2:**
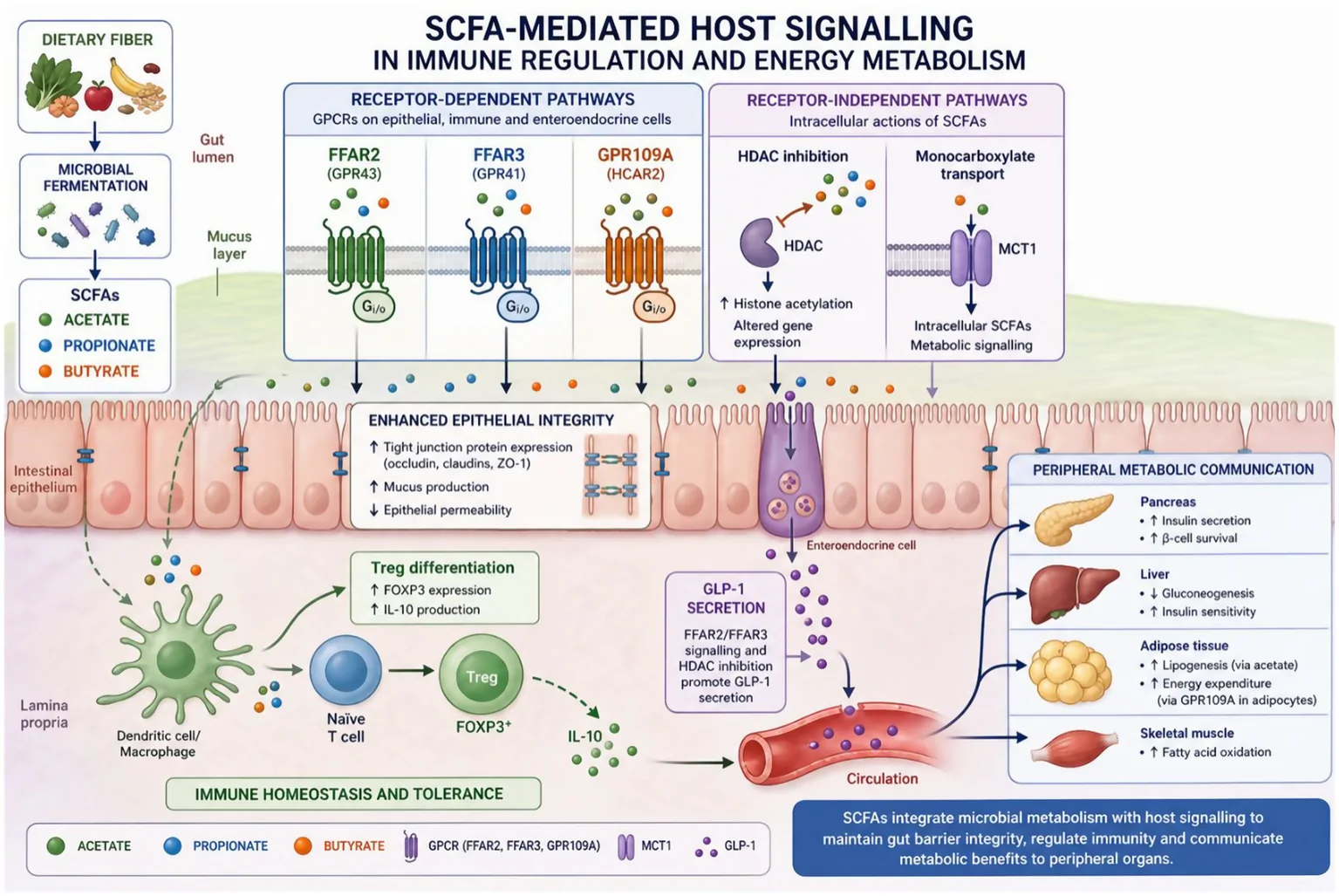
SCFA-mediated host signaling in immune regulation and energy metabolism. The figure illustrates receptor-dependent (FFAR2, FFAR3, GPR109A) and receptor-independent (HDAC inhibition, monocarboxylate transport) pathways through which acetate, propionate, and butyrate regulate epithelial integrity, regulatory T-cell differentiation, enteroendocrine hormone secretion, and peripheral metabolic communication.

### 5.5 Branched-chain fatty acids as emerging complementary metabolites

In addition to acetate, propionate, and butyrate, microbial fermentation produces branched-chain fatty acids (BCFAs), such as isobutyrate, isovalerate, and 2-methylbutanoate. These metabolites are predominantly produced by bacterial fermentation of branched-chain amino acids (valine, leucine, and isoleucine) and become more prominent in situations of enhanced protein fermentation. Although BCFAs make up a small proportion of total colonic fatty acids, new research suggests they may play important roles in epithelial signaling, mineral transport, and localized immunological modulation (Mao et al., 2023; Mukhopadhya and Louis, 2025). However, mechanistic and clinical evidence for BCFAs remains sparse compared with that for classical SCFAs. As a result, BCFAs should be viewed as potential but understudied microbial metabolites that could serve as indicators of altered fermentative balance and microbial ecology.

### 5.6 Dose-dependent and bidirectional effects of SCFAs

The biological effects of SCFAs are influenced by SCFA type, concentration, exposure duration, tissue, and cellular metabolic state, which may explain apparently conflicting findings. Acetate is the predominant circulating SCFA; propionate is preferentially taken up by the liver for gluconeogenesis and lipid metabolism, whereas butyrate is primarily oxidized by colonocytes (Boets et al., 2017; Den Besten et al., 2013; Portincasa et al., 2022). Thus, luminal SCFA concentrations cannot be directly equated with systemic exposure or biological effects.

At physiological exposure levels, SCFAs generally contribute to epithelial integrity, metabolic homeostasis, and immune regulation through FFAR2/FFAR3 signaling, cellular metabolism, and HDAC inhibition. However, their effects may become concentration- and context-dependent at higher exposure levels. Butyrate and propionate can promote regulatory T-cell responses and limit inflammation under appropriate conditions, whereas higher concentrations may induce cellular stress or pro-inflammatory signaling in specific immune contexts (Mirmonsef et al., 2012; Parada Venegas et al., 2019).

The butyrate paradox further illustrates this bidirectional behavior. Butyrate serves as an oxidative fuel for normal colonocytes and supports epithelial homeostasis, whereas increased intracellular butyrate can inhibit proliferation and induce apoptosis in colorectal cancer cells through HDAC inhibition. This difference is partly attributed to the Warburg phenotype of cancer cells, which reduces butyrate oxidation and promotes its intracellular and nuclear accumulation (Donohoe et al., 2012). Thus, apparently conflicting proliferative and antiproliferative effects may reflect differences in concentration, cellular metabolic phenotype, exposure duration, and experimental conditions.

Overall, SCFAs should be viewed as context-dependent metabolic signals rather than inherently beneficial or harmful metabolites. Their effects can be interpreted as interactions among SCFA species × concentration × exposure duration × tissue/cell type × metabolic state. Future prebiotic studies should therefore distinguish individual SCFA concentrations and tissue exposure from total SCFA production and integrate these measurements with host metabolic and immune responses.

## 6 Experimental models for host–microbe interactions

Direct investigation of prebiotic fermentation and microbiota-derived metabolite synthesis in humans is hampered by limited access to intestinal sampling sites, high inter-individual variability, and ethical constraints associated with repeated gastrointestinal sampling. As a result, *in vitro* gastrointestinal fermentation systems have emerged as valuable preclinical tools for investigating microbial substrate utilization, short-chain fatty acid (SCFA) production, and host–microbe interactions under controlled experimental conditions (Venema and Van den Abbeele, 2013; Van den Abbeele et al., 2013).

In contrast to *in vivo* research, these methods enable parallel assessment of several dietary fibers and donor microbiomes while providing exact control over pH, substrate composition, anaerobic conditions, and sample intervals. Mechanistic and translational microbiome research is facilitated by the growing integration of contemporary fermentation platforms with metagenomics, metabolomics, and computational modeling techniques (Singh et al., 2022; Van den Abbeele et al., 2013).

Importantly, advanced *in vitro* models can replicate many microbial fermentation patterns and metabolite profiles found in the human colon while retaining significant microbial diversity derived from human fecal inocula (Venema and Van den Abbeele, 2013; Van den Abbeele et al., 2013; Singh et al., 2022). These systems can also support key fiber-fermenting and SCFA-associated taxa such as *Bifidobacterium*, *Faecalibacterium*, *Roseburia*, *Akkermansia*, *Bacteroides*, and *Prevotella*, making them useful platforms for assessing microbiome functionality and responsiveness to dietary interventions (Venema and Van den Abbeele, 2013; Van den Abbeele et al., 2013; Davani-Davari et al., 2019).

### 6.1 Dynamic *in vitro* fermentation models

. Static batch fermentation systems remain the most widely used methods for assessing fiber degradability, microbial compositional changes, gas production, and SCFA synthesis. They are useful screening platforms for comparing several substrates at once because of their ease of use, affordability, and high throughput capacity (Riva et al., 2023; Payne et al., 2012). However, their static nature limits the simulation of continuous nutrition flow, microbial adaptability, and regional gastrointestinal compartmentalization.

Continuous and multi-compartment fermentation models have been created to better simulate the dynamic circumstances found in the human gut. These systems allow for greater control over physicochemical conditions and the long-term culture of complex microbial populations, resulting in a more physiologically relevant environment for microbial fermentation. One of the most extensively characterized platforms is the Simulator of the Human Intestinal Microbial Ecosystem (SHIME), which replicates sequential gastrointestinal compartments and allows for the study of region-specific fermentation dynamics, microbial metabolism, and host-relevant metabolite production (Zhu et al., 2024). Figure 3. Schematic representation of the SHIME® continuous and multi-compartment fermentation model for SCFA and metabolite production.

**Figure 3:**
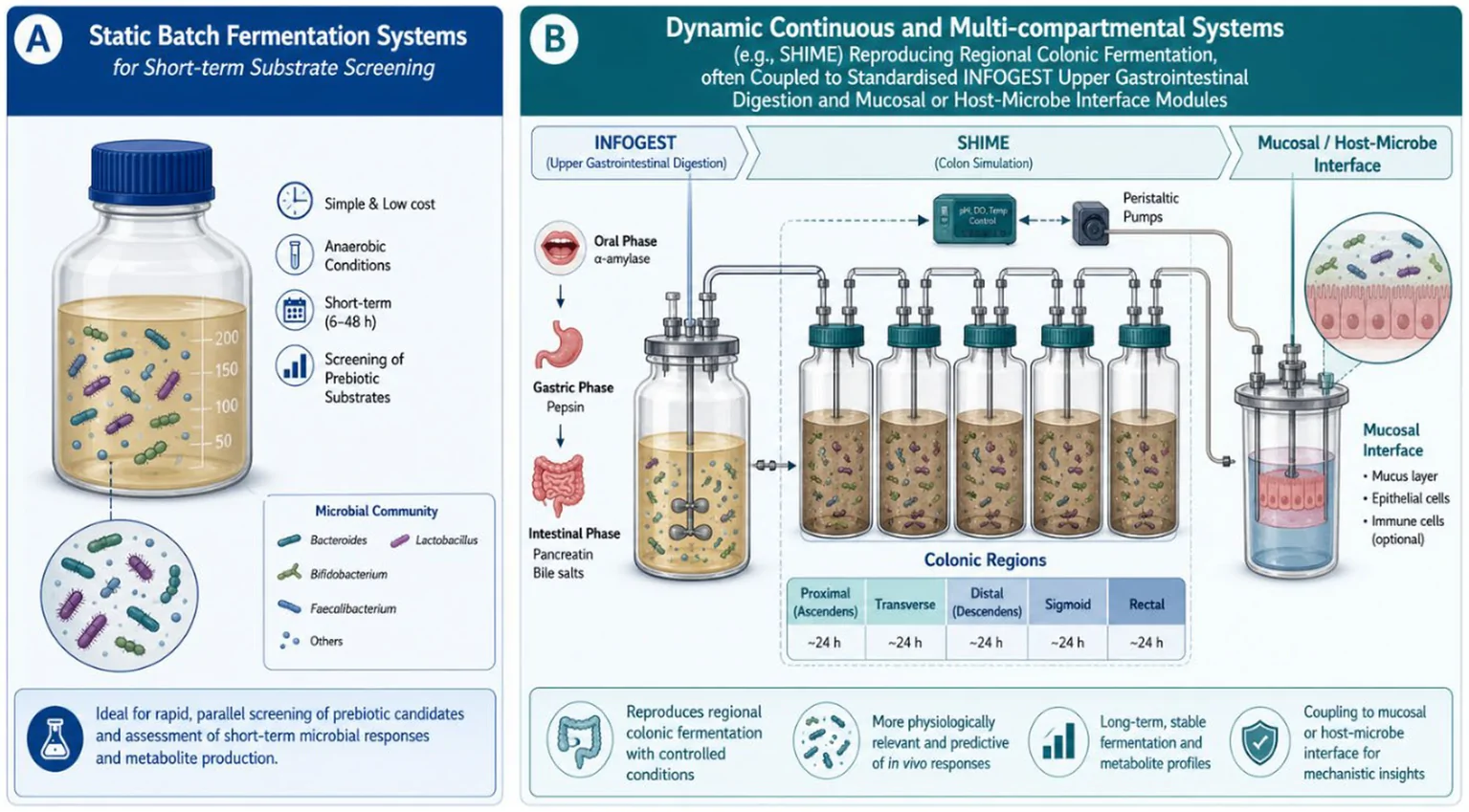
*In vitro* fermentation systems used in prebiotic research. **(A)** Static batch fermentation systems for short-term substrate screening. **(B)** Dynamic continuous and multi-compartmental systems (e.g., SHIME) reproducing regional colonic fermentation, often coupled to standardized INFOGEST upper gastrointestinal digestion and mucosal or host-microbe interface modules.

### 6.2 Mucosal and host–microbe interface innovations

The absence of epithelial contact and an intact mucus layer represents a significant limitation of conventional fermentation systems. Recently developed models have addressed this limitation by incorporating artificial mucus matrices and mucosal simulation platforms that more accurately reproduce microbial colonization of the gut surface and host–microbe interactions (Calvigioni et al., 2023; Villegas-Novoa et al., 2024). These advancements have led to the integration of microbial fermentation with models of epithelial, immunological, and enteroendocrine cells in sophisticated experimental systems. These platforms allow evaluation of barrier integrity, cytokine responses, transporter activity, and hormone secretion following exposure to microbiota-derived compounds (Hill and Spence, 2017; Lee et al., 2024). Emerging gut-on-a-chip and intestinal organoid technologies increase physiological relevance by replicating intestinal fluid dynamics, oxygen gradients, cellular differentiation, and aspects of tissue architecture that are difficult to capture in traditional fermentation systems (Hill and Spence, 2017; Villegas-Novoa et al., 2024; Lee et al., 2024).

### 6.3 Personalized and high-throughput fermentation platforms

Growing interest in precision nutrition has stimulated the development of donor-specific fermentation systems that evaluate identical substrates across multiple human microbiomes. Poeker et al. (2018) and Nogacka et al. (2020) found significant inter-individual variability in microbial composition, fermentation capacity, gas output, and SCFA generation. Such findings emphasize the necessity of individualized prebiotic interventions and facilitate the identification of individuals who are more likely to respond strongly or weakly to particular substrates. Automated culture techniques and high-throughput bioreactor arrays now allow numerous substrates and microbial populations to be screened simultaneously under standardized conditions. When combined with metabolomics, computational modeling, and machine learning, these platforms offer new opportunities for predicting individual responses to dietary interventions and identifying microbial features associated with responder and non-responder phenotypes (Deehan et al., 2020; Riva et al., 2023; Kolodziejczyk et al., 2019).

### 6.4 Standardized *in vitro* gastrointestinal digestion models

Beyond substrate composition itself, structural modifications that occur during digestion can significantly influence the accessibility of dietary substrates to the gut microbiota. To simulate oral, gastric, and intestinal processing prior to colonic fermentation analysis, standardized *in-vitro* digestion protocols have been developed (Mulet-Cabero et al., 2020). The most widely adopted framework is currently the INFOGEST consensus model, which provides standardized experimental conditions for pH, transit time, bile salt concentrations, and enzyme exposure, thereby enhancing comparability across laboratories (Brodkorb et al., 2019; Le Feunteun et al., 2020). Integrating these standardized digestion procedures with downstream fermentation models enables a more realistic assessment of microbiota-accessible substrates available for colonic fermentation and improves the reproducibility and physiological relevance of experimental outcomes.

### 6.5 Current strengths, translational limitations, and strategies for improvement

Modern *in vitro* gut models provide controlled and reproducible systems for investigating microbial substrate utilization, fermentation dynamics, SCFA production, and host–microbe interactions. They are particularly useful when combined with ‘multi-omics’ approaches (Payne et al., 2012; Van den Abbeele et al., 2013). However, their translation to human physiology is limited by three major gaps: incomplete mucosal interactions, inadequate representation of systemic responses, and limited simulation of intestinal dynamics.

First, conventional fermentation systems lack mucus and direct epithelial–immune interactions, limiting their ability to reproduce mucosal colonization, barrier function, and immune signaling (Calvigioni et al., 2023; Hill and Spence, 2017). Artificial mucus matrices, epithelial co-culture, and immune-cell incorporation can improve these models. Second, most systems lack vascular and systemic compartments and therefore poorly represent SCFA absorption, hepatic metabolism, and peripheral signaling. Perfused epithelial–vascular interfaces and organ-on-chip platforms may better simulate metabolite transport and systemic exposure (Hill and Spence, 2017; Lee et al., 2024). Third, static models inadequately reproduce peristalsis, fluid flow, nutrient gradients, and regional oxygen and pH variation. Dynamic fluid perfusion and mechanically active gut-on-chip systems can improve physiological relevance (Lee et al., 2024).

Thus, integrating artificial mucus/epithelial co-culture, immune-cell incorporation, and dynamic perfusion, together with standardized digestion, donor-specific microbiomes, metabolomics, and computational modeling, may strengthen the predictive value of *in vitro* platforms for clinical outcomes. Nevertheless, these systems remain mechanistic intermediary models requiring validation in human studies.

## 7 Translational and clinical evidence

The potential uses of prebiotic-induced SCFA synthesis in metabolic, inflammatory, neoplastic, and neurophysiological conditions have raised considerable interest. Human intervention studies show that specific prebiotic supplementation can modify gut microbial composition, enhance saccharolytic fermentation, alter SCFA production, and improve selected disease-related or physiological outcomes. However, the magnitude and consistency of these effects vary substantially according to baseline microbiota composition, habitual dietary fiber intake, intervention duration, medication exposure, gastrointestinal tolerance, and disease status (Müller et al., 2018; Nogacka et al., 2020). An important consideration in interpreting clinical SCFA studies is that fecal SCFA concentrations do not directly represent total microbial SCFA production or systemic SCFA availability. Fecal concentrations reflect the balance among microbial production, intestinal absorption, epithelial and hepatic metabolism, and fecal excretion. Consequently, changes in fecal SCFA concentrations should be interpreted together with microbiota composition, substrate utilization, host metabolic responses, and, where available, circulating SCFA measurements rather than being considered as a direct surrogate of whole-body SCFA exposure. Representative human clinical studies evaluating prebiotic supplementation, dosage, microbiota changes, SCFA responses, and clinical outcomes are summarized in Table 4.

**Table 4:** Representative human clinical studies evaluating prebiotic supplementation, dosage, microbiota changes, SCFA production, and clinical outcomes.

Prebiotic type	Study design	Dose/duration	Major microbiota changes	SCFA response	Principal clinical outcomes	References
Inulin-type fructans	RCT, double-blind placebo-controlled; adults with type 2 diabetes (*n* = 42)	16 g/day for 6 weeks	Significant enrichment of *Bifidobacterium adolescentis* and Bacteroides-associated OTUs	Increased total fecal SCFAs, particularly acetate and propionate	Improved gut microbiota composition and enhanced fermentative activity in type 2 diabetes	Birkeland et al. (2020)
Galactooligosaccharides (GOS)	Double-blind placebo-controlled crossover; healthy adults (*n* = 44)	5.5 g/day for 10 weeks	Significant increase in bifidobacteria abundance	Not specifically quantified; indirect evidence of enhanced saccharolytic fermentation	Increased phagocytic activity, enhanced NK cell function, increased IL-10, and reduced IL-6, IL-1β, and TNF-α	Vulevic et al. (2015)
Resistant starch type II (potato starch)	Randomized crossover; adults with metabolic syndrome (*n* = 20)	12 g- 48 g/day for 3 weeks	Increased abundance of resistant starch degraders, particularly *Bifidobacterium adolescentis* and *Ruminococcus bromii*	Mean fecal butyrate increased by ~67%	Strong inter-individual microbiome-dependent metabolic responsiveness	Venkataraman et al. (2016)
Xylooligosaccharides (XOS)	RCT double-blind, placebo-controlled; healthy adults (*n* = 32)	1.4 or 2.8 g/day for 8 weeks	Dose-dependent increase in *Bifidobacterium* spp., strongest at 2.8 g/day	No significant change in total fecal SCFA levels	Selective bifidogenic effect without parallel lactobacilli increase	Finegold et al. (2014)
Mixed fiber supplements (FOS, CAX, SAX)	Controlled donor fecal fermentation intervention; healthy adults (*n* = 6)	15 g/day equivalent substrate exposure for 6 weeks	Enterotype-dependent stimulation of *Prevotella* OTUs	High total SCFA generation, with propionate as the dominant product in *Prevotella* enterotype	Donor microbiome-dependent differences in fiber utilization and SCFA profile	Chen et al. (2017)
Human milk oligosaccharides (HMOs)	Oral dose-escalation intervention; healthy adults (*n* = 32)	1.8–18 g/day for 7 days + 21-day follow-up	Dose-dependent expansion of bifidobacteria with reduced overall microbial diversity	Increased acetate and butyrate concentrations	Supports HMO use as a microbiota-directed therapeutic strategy in dysbiosis-associated conditions	Jacobs et al. (2023); Lordan et al. (2024)
Red wine polyphenols	Randomized controlled crossover; metabolic syndrome and healthy adults (*n* = 20)	30-day intervention after washout	Increased fecal bifidobacteria, lactobacilli, *Faecalibacterium prausnitzii*, and *Roseburia*	Indirect butyrogenic shift inferred from enrichment of butyrate producers	Beneficial microbiota remodeling relevant to obesity-associated metabolic dysfunction	Moreno-Indias et al. (2016)

The included clinical studies encompass heterogeneous participant populations, ranging from healthy adults to individuals with metabolic dysfunction or related disorders. Reported microbiota, SCFA, and clinical outcomes should be interpreted in relation to baseline metabolic phenotype, disease status, habitual diet, and study-specific intervention conditions. Direct quantitative comparisons across studies should be approached with caution because of differences in participant characteristics, substrate dose, intervention duration, analytical methodology, and endpoint selection.

RCT, randomized controlled trial; NK, natural killer; IL, interleukin; OTU, operational taxonomic unit; CAX, corn arabinoxylan; SAX, sorghum arabinoxylan.

### 7.1 Type 2 diabetes, obesity, and cardiometabolic dysfunction

Type 2 diabetes and obesity are among the most extensively studied therapeutic targets for prebiotic intervention. Fermentable fibers, including resistant starche, galactooligosaccharides, and inulin-type fructans may improve glycemic control and cardio metabolic regulation. Meta-analyses of supplementation with inulin-type carbohydrates have shown reduction in fasting glucose, insulin, HbA1c, and HOMA-IR, especially in people with metabolic dysfunction and low baseline fiber intake (Rao et al., 2019; Birkeland et al., 2020). Increased generation of SCFA, improved gut barrier function, reduced endotoxaemia, stimulation of GLP-1 secretion, and regulation of chronic low-grade inflammation are among the suggested mechanisms (Canfora et al., 2019; Morrison and Preston, 2016). Despite encouraging findings, clinical outcomes remain inconsistent, with some studies demonstrating meaningful metabolic benefits and others reporting only modest microbiota alterations. Consequently, prebiotics are currently regarded as promising adjunctive, rather than stand-alone, strategies for metabolic disease management.

### 7.2 Inflammatory bowel disease and intestinal inflammation

Clinical trials of fructooligosaccharides, inulin-type fructans, and galactooligosaccharides in patients with IBD have reported enrichment of beneficial microbial groups and changes in inflammatory markers and disease-related parameters; however, findings remain inconsistent across populations and intervention types (Wilson et al., 2021). Individual responsesvary substantially. Some patients show changes in the microbiome and inflammatory markers, whereas others experience only partial symptom improvement. Moreover, fermentable fibers are not well tolerated by all patients, and susceptible individuals may experience symptoms such as abdominal pain or bloating (Akutko and Stawarski, 2021; Roy and Dhaneshwar, 2023). Prebiotic compounds may therefore be most appropriate as adjunctive interventions during remission or mild disease (Akutko and Stawarski, 2021).

### 7.3 Colorectal neoplasia and colonic protection

One promising application of SCFA-mediated microbiota modification is the prevention of colorectal cancer. Depletion of butyrate-producing taxa such as *Roseburia* and *Eubacterium* is commonly observed in colorectal neoplasia and observational studies consistently associate higher intake of fermentable fibers with reduced colorectal cancer risk (Sędzikowska and Szablewski, 2021; Pedrosa and Fabi, 2024). Mechanistic studies indicate that butyrate affects immune surveillance, oxidative stress reactions, apoptosis, epithelial differentiation, and epigenetic regulation in cancerous tissues. The translational promise of microbiota-directed therapeutics is further highlighted by new approaches, such as colon-targeted prebiotic–drug delivery systems (Zeybek et al., 2023). Nevertheless, long-term randomized controlled trials demonstrating that prebiotic supplementation alone reduces colorectal cancer incidence or recurrence remain limited. Prebiotics should therefore be regarded as components of broader colorectal-protective dietary strategies rather than validated chemo preventive monotherapies (Liu et al., 2024).

### 7.4 Gut–brain axis and neuropsychiatric applications

An increasingly significant field of translational research is the gut-brain axis. Experimental findings indicate that prebiotic-induced microbial and metabolic alterations may influence neuroimmune pathways, stress reactions, and neuroendocrine signaling through interrelated immune, endocrine, and neurological systems (Savignac et al., 2013; Ullah et al., 2023). Modest benefits in stress perception, anxiety-related features, sleep quality, and certain cognitive outcomes have been documented in human trials employing fructooligosaccharides and galactooligosaccharides. However, due to variations in study design, participant characteristics, and outcome assessment techniques, results continue to be inconsistent (Chudzik et al., 2021; Chakrabarti et al., 2022; Nikolova et al., 2021). As a result, the neuropsychiatric applications of prebiotics should currently be regarded as promising but exploratory and require further confirmation in larger longitudinal studies that include metabolomic, neurobehavioral, and microbiome assessments (Chakrabarti et al., 2022; Ullah et al., 2023).

### 7.5 Operable stratified framework for precision prebiotic nutrition

The substantial inter-individual variability in response to prebiotic interventions suggests that a uniform substrate and dose may not be appropriate for all individuals. Baseline microbiota, habitual dietary fiber intake, medication exposure, and host metabolic status can instead be integrated into a stratified responder framework to guide prebiotic selection and intervention intensity. Studies show that baseline gut microbial characteristics and habitual fiber intake can influence responses to resistant starch and other fermentable fibers. Similarly, the response to inulin varies significantly among individuals (Devarakonda et al., 2024; Van-Wehle and Vital, 2024).

A practical framework can be based on four domains: (i) microbiome-mediated fermentation capacity, (ii) habitual dietary fiber exposure, (iii) host metabolic phenotype, and (iv) medication and clinical context. Microbiome assessment should consider both taxonomic abundance and functional potential. The abundance of butyrate-associated taxa, including *Faecalibacterium*, *Roseburia*, *Anaerostipes*, and *Agathobacter*, may provide an initial indication of fermentation capacity. However, substrate-specific responses indicate that microbial composition alone does not guarantee functional SCFA production. Different fibers can stimulate distinct microbial populations, highlighting the importance of substrate–microbiota compatibility.

Habitual fiber intake should include total fiber consumption, major fiber sources, resistant-starch exposure, and diversity of plant-derived substrates. Individuals with low habitual fiber intake may have greater scope for adaptation, whereas those already consuming diverse fermentable fibers may show a smaller incremental response to a single substrate. Host metabolic phenotype, including glucose regulation, insulin sensitivity, adiposity, lipid status, and low-grade inflammation, should inform the therapeutic objective. Prebiotic supplementation should remain an adjunct to established dietary and lifestyle management rather than a replacement for standard treatment.

Medication exposure and clinical context should also be considered because medications, particularly antibiotics, can alter microbiota composition and intestinal physiology. These factors should therefore be recorded as potential covariates or stratification variables when interpreting intervention responses.

Based on these domains, individuals can be provisionally classified as high-responder, low/intermediate-responder, or non-responder/intolerant profiles (Table 5). High responders may have favorable fermentation capacity, compatible microbiota, relatively low-to-moderate habitual fiber exposure, and good gastrointestinal tolerance. Low/intermediate responders may have moderate fermentation capacity, high exposure to similar substrates, or partial substrate–microbiota compatibility; gradual dose escalation, mixed-fiber strategies, or alternative substrates may therefore be appropriate. Non-responders or intolerant individuals may have limited capacity to utilize the selected substrate or develop significant gastrointestinal symptoms. Increasing the same substrate dose may be inappropriate, and modification or discontinuation should be considered.

**Table 5:** Simplified stratified framework for precision prebiotic intervention.

Stratification domain	High responder	Low/intermediate-responder	Non-responder/intolerant	Potential intervention
Butyrate-producing capacity	Relatively high abundance/function of *Faecalibacterium, Roseburia, Anaerostipes, Agathobacter, and Eubacterium rectale* (Louis and Flint, 2009; Liu et al., 2023)	Moderate abundance or incomplete functional capacity	Very low/limited relevant functional potential	Match substrate to microbiome capacity; consider resistant starch or compatible substrates (Martinez et al., 2010; Devarakonda et al., 2024)
Substrate-degrading network	Well-established substrate utilization and cross-feeding	Moderate or incomplete network	Limited ability to utilize substrate	Introduce gradually or consider an alternative/mixed-fiber substrate (Martinez et al., 2010; Devarakonda et al., 2024)
Habitual fiber intake	Low–moderate intake with adaptation potential	Moderate–high intake	High prior exposure with little additional response	Start conservatively and avoid unnecessary dose escalation (Healey et al., 2018; Holmes et al., 2022)
Metabolic phenotype	Clear metabolic target and stable status	Mild metabolic dysfunction	Advanced/unstable disease or competing factors	Match intervention intensity to clinical needs and standard care (Gibson et al., 2017; Holmes et al., 2022)
Medication/clinical context	No major microbiome-disrupting exposure	Stable medication use	Recent antibiotics or major clinical instability	Record as covariates and consider timing/stabilization (Zimmermann and Curtis, 2019)
Initial substrate strategy	Targeted, compatible substrate	Low starting dose with gradual increase	Alternative substrate or reassessment	Individualize substrate and dose according to capacity and tolerance (Gibson et al., 2017; Devarakonda et al., 2024)
Response monitoring	Clinical benefit and good tolerance	Partial benefit or mild GI effects	No benefit or marked intolerance	Continue, modify, switch, or discontinue according to response (Martinez et al., 2010; Holmes et al., 2022)
Reassessment	Maintain or optimize	Reassess substrate and dose	Reclassify and avoid escalation	Repeat assessment and modify intervention based on longitudinal response (Holmes et al., 2022; Devarakonda et al., 2024)

These categories should be viewed as dynamic intervention phenotypes rather than fixed biological traits because microbiota composition, diet, medication use, disease activity, and host metabolism can change over time. An individual who responds poorly to one substrate may respond to another with a different structural composition.

Accordingly, precision prebiotic intervention can follow five stages: baseline phenotyping, responder classification, substrate matching, controlled dose escalation, and response reassessment. Baseline assessment should include habitual dietary fiber intake and substrate exposure, relevant clinical and metabolic characteristics, medication exposure, gastrointestinal tolerance, and, where feasible, microbiome composition or functional capacity and SCFA-related measurements. A compatible prebiotic can then be introduced at a low-to-moderate dose and gradually increased according to tolerance. Response should be reassessed using clinical outcomes, gastrointestinal symptoms, microbial changes, and metabolic or SCFA-related biomarkers rather than fecal SCFA concentrations alone, because fecal SCFA concentrations reflect the balance among microbial production, intestinal absorption, host metabolism, and excretion.

The proposed framework is summarized in Table 5 and provides an operational approach for linking measurable baseline characteristics with differentiated intervention strategies. However, the responder categories and decision rules remain hypothesis-generating rather than clinically validated algorithms. Prospective studies integrating microbiome profiling, dietary assessment, metabolomics, medication exposure, and host metabolic phenotyping are needed to establish reproducible thresholds and externally validated prediction models.

## 8 Current challenges and knowledge gaps

Despite rapid advances in microbiome research, several challenges continue to limit the translation of prebiotic interventions into evidence-based nutritional practice. Human studies remain highly heterogeneous with respect to prebiotic type, dosage, intervention duration, dietary background, microbiome assessment methods, and clinical outcomes, complicating comparisons across studies and reducing the strength of pooled evidence (Vinelli et al., 2022).

One of the major limitations in this field is the dependence on surrogate biomarkers, particularly fecal short-chain fatty acid concentrations, to assess microbial fermentation activity. Although widely used, fecal SCFA measurements provide only an indirect estimate of microbial metabolic activity and may not accurately reflect actual SCFA production within the colon or systemic SCFA exposure. Interpretation of these measurements is further complicated by factors such as sample collection and storage conditions, transit time, dietary influences, and inter-individual differences in SCFA absorption and utilization. In addition, methodological variability among analytical techniques, including gas chromatography (GC), liquid chromatography–mass spectrometry (LC–MS), high-performance liquid chromatography (HPLC), and nuclear magnetic resonance (NMR) spectroscopy, can affect the comparability of results across studies (Han et al., 2015; Kim et al., 2022).

Substantial inter-individual variation persists even when similar health benefits are targeted through prebiotic interventions. Fermentation efficiency and responses to prebiotic supplementation can be influenced by baseline gut microbiota composition, dietary practices, medication use, host physiological characteristics, immune status, and genetic background. Consequently, clinical outcomes can differ substantially among participants in intervention studies (Holmes et al., 2022; Van-Wehle and Vital, 2024).

*In vivo* models, *in vitro* fermentation systems, organoids, and host–microbe interaction models have substantially advanced mechanistic understanding. However, findings from these models cannot always be directly extrapolated to human physiology. Differences in microbial composition, immune function, host metabolism, and environmental exposures can contribute to discrepancies between experimental findings and clinical outcomes. In addition, inconsistencies in DNA sequencing technologies, bioinformatics pipelines, and statistical approaches can reduce reproducibility across studies and complicate the development of robust, broadly applicable microbiome biomarkers.

Collectively, these limitations highlight the need for longitudinal human studies, standardized study designs, validated biomarkers, and harmonized analytical methods to support reproducible and clinically effective microbiota-targeted dietary strategies.

## 9 Discussion and future perspectives

The evidence reviewed indicates that prebiotic efficacy cannot be explained by substrate classification, microbiota composition, SCFA production, or host responses independently. Rather, biological effects emerge through a continuum linking prebiotic molecular structure, microbial functional capacity, SCFA metabolism, host signaling, and individual response. Accordingly, we propose a three-layer framework integrating (i) the prebiotic structure–microbial CAZyme/PUL–SCFA metabolic axis, (ii) SCFA-mediated immune and metabolic signaling, and (iii) stratified precision prebiotic intervention.

### 9.1 Layer 1: prebiotic structure–microbial CAZyme–SCFA axis

Structural characteristics such as degree of polymerization, glycosidic linkage, branching, solubility, accessibility, and resistance to digestion influence substrate delivery to the colon. Consequently, classification as a prebiotic does not necessarily predict metabolic responses across individuals. Utilization depends partly on the presence and activity of carbohydrate-active enzymes (CAZymes), polysaccharide utilization loci (PULs), transport systems, and associated metabolic pathways. Microbial cross-feeding further influences SCFA production, meaning that acetate, propionate, and butyrate synthesis represents an emergent property of microbial metabolic networks rather than the activity of individual taxa. Future studies should therefore integrate metagenomics, metatranscriptomics, substrate utilization, and metabolomics to distinguish functional potential from actual metabolic activity.

### 9.2 Layer 2: SCFA–host signaling

The second layer connects microbial SCFA production with host physiology. SCFAs influence epithelial integrity, immune regulation, endocrine signaling, and systemic metabolism. These effects can be conceptually organized into interconnected immune–epithelial and metabolic–endocrine pathways involving mechanisms such as histone deacetylase inhibition and activation of FFAR2/FFAR3. However, SCFA effects are context dependent and influenced by concentration, tissue exposure, receptor distribution, metabolic state, and disease status. Importantly, fecal SCFA concentrations alone do not necessarily reflect systemic SCFA signaling or clinical benefit.

### 9.3 Layer 3: stratified precision prebiotic nutrition

The third layer translates these mechanisms into individualized intervention. Individuals may be provisionally categorized as high-, low/intermediate-, or non-responder/intolerant profiles according to microbial functional capacity, dietary history, host phenotype, and gastrointestinal tolerance. These categories should remain dynamic rather than permanent because microbiota and dietary exposures can change. A potential strategy is baseline characterization → functional stratification → substrate matching → controlled dose escalation → microbiome/metabolite and clinical assessment → intervention adjustment.

### 9.4 Translational perspectives

The three layers form a continuous chain: substrate structure - CAZyme/PUL-mediated utilization - microbial cross-feeding - SCFA production - host signaling - physiological response – substrate –dose matching. Future trials should therefore employ defined substrates, standardized methods, controlled dietary backgrounds, appropriate comparators, and longitudinal multi-omics and host phenotyping. Computational and machine-learning approaches may improve prediction of nonlinear interactions between substrate, microbiota, metabolism, and host response (Holmes et al., 2022; Wang et al., 2025). Nevertheless, the proposed responder framework remains hypothesis-generating rather than a validated clinical algorithm and requires prospective validation across diverse populations. Emerging approaches, including colon-targeted delivery, food-matrix modification, synbiotics, and postbiotics, may further improve efficacy and tolerance (Manzari et al., 2021; Azehaf et al., 2023). Overall, precision prebiotic nutrition should move beyond the “one substrate–one population” model toward an adaptive strategy integrating substrate structure, microbial functional capacity, SCFA metabolism, host signaling, and individual response.

## 10 Conclusion

Prebiotic fibers represent an important dietary strategy for modulating the gut microbiota and microbial SCFA production. Through the selective stimulation of beneficial microbes and cross-feeding interactions, prebiotics influence SCFA-mediated pathways involved in epithelial maintenance, immune regulation, endocrine signaling, and metabolic homeostasis. Although mechanistic evidence supporting these effects is substantial, translation into reproducible clinical benefits remains limited by inter-individual variability. Accordingly, increasing attention is being directed toward integrative approaches combining microbiome research, multi-omics analyses, computer modeling, and precision nutrition. Such approaches may enable more precise characterization and prediction of individual response and improve our understanding of diet-microbiome interactions. Prebiotics therefore remain biological relevant tools for investigating and potentially modulating host–microbe interactions. Future progress will require standardized method, rigorous clinical validation, and greater integration across microbiology, nutrition, and systems biology.
